# Enterovirus and Parechovirus Surveillance — United States, 2014–2016

**DOI:** 10.15585/mmwr.mm6718a2

**Published:** 2018-05-11

**Authors:** Glen R. Abedi, John T. Watson, W. Allan Nix, M. Steven Oberste, Susan I. Gerber

**Affiliations:** 1 Division of Viral Diseases, National Center for Immunization and Respiratory Disease, CDC.

Infections caused by enteroviruses (EV) and parechoviruses (PeV), members of the Picornaviridae family, are associated with various clinical manifestations, including hand, foot, and mouth disease; respiratory illness; myocarditis; meningitis; and sepsis; and can result in death. The genus *Enterovirus* includes four species of enterovirus (A–D) known to infect humans, and the genus *Parechovirus* includes one species (A) that infects humans. These species are further divided into types, some of which are associated with specific clinical manifestations. During 2014–2016, a total of 2,967 U.S. cases of EV and PeV infections were reported to the National Enterovirus Surveillance System (NESS). The largest number of reports during that time (2,051) occurred in 2014, when a large nationwide outbreak of enterovirus D68 (EV-D68) occurred, accounting for 68% of cases reported to NESS that year (*1*). Reports to the National Respiratory and Enteric Virus Surveillance System (NREVSS) during 2014–2016 indicated that circulation of EV peaks annually in the summer and early fall. Because the predominant types of EV and PeV circulating from year to year tend to vary, tracking these trends requires consistent and complete reports from laboratories with the capacity to perform typing.

NESS is a passive, laboratory-based surveillance system that has been used to track EV and PeV reports since the 1960s and is the most comprehensive database of these reports in the United States. During 2014–2016, 11 laboratories reported to NESS, including nine state health departments, one municipal health department, and the CDC Polio and Picornavirus Laboratory Branch (PPLB). The largest contributor of reports to NESS was PPLB (1,553), which serves as a reference laboratory for jurisdictions with no or limited EV and PeV typing capacity. Testing data for untyped EV are also collected through NREVSS, a passive, laboratory-based surveillance system that collects aggregate reports of tests for EV and the percentage positive by week.

During 2014–2016, a total of 2,967 EV and PeV cases were reported to NESS, including 2,758 (93.0%) for which the type was known. Reports that included virus type represented 2,734 individual patients, among whom one virus type was identified from 2,726 (99.7%) and two types were identified from eight (0.3%). Among 2,370 (86.7%) patients with known sex, 1,422 (60.0%) were male, and among 1,351 (90.1%) for whom age was known, the median age was 4 years (interquartile range = 1–10 years). State of residence was known for 2,727 (99.7%) patients; among these, California was the most frequently reported state (413, 15.1%), followed by New York (366, 13.4%). Residents from all 50 states and the District of Columbia were represented (Figure 1). The largest number of reports to NESS that included EV and PeV type (2,051) occurred in 2014 (Box); these reports accounted for 74% of the 2,758 reports for all 3 years.

**FIGURE 1:**
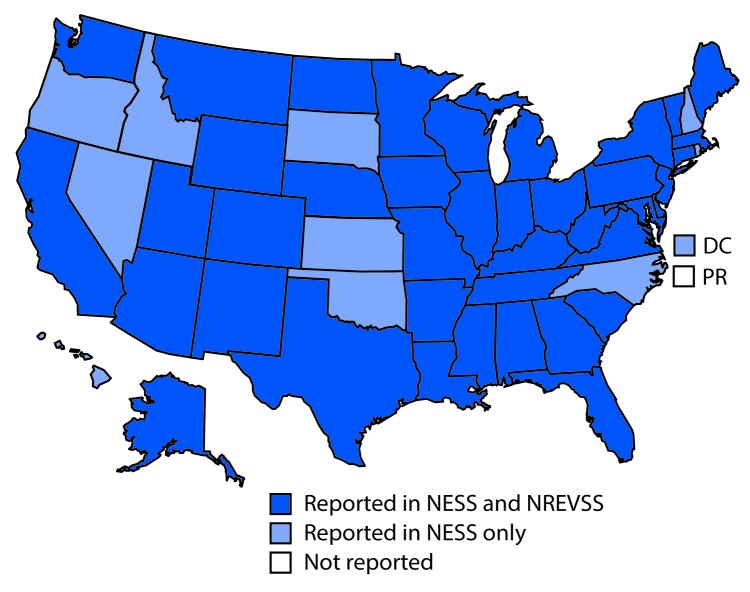
States from which enterovirus-positive or parechovirus-positive results were reported, by surveillance system — United States, 2014–2016 **Abbreviations:** DC = District of Columbia; NESS = National Enterovirus Surveillance System; NREVSS = National Respiratory and Enteric Virus Surveillance System; PR = Puerto Rico.

BOX **Distribution of the 15 enterovirus and human parechovirus types most frequently reported, by year — National Enterovirus Surveillance System, United States, 2014–2016**

**Table:** 

2014 (N = 2,051)		2015 (N = 370)		2016 (N = 337)		2014–2016 (N = 2,758)	
Type	No. (%)	Type	No. (%)	Type	No. (%)	Type	No (%)
Enterovirus D68	1,395 (68.0)	Echovirus 30	100 (27.0)	Enterovirus D68	138 (40.9)	Enterovirus D68	1,542 (55.9)
Coxsackievirus B3	98 (4.8)	Echovirus 18	61 (16.5)	Coxsackievirus A6	39 (11.6)	Echovirus 30	159 (5.8)
Coxsackievirus A6	86 (4.2)	Coxsackievirus A6	27 (7.3)	Coxsackievirus B4	18 (5.3)	Coxsackievirus A6	152 (5.5)
Echovirus 11	52 (2.5)	Echovirus 3	21 (5.7)	Echovirus 6	15 (4.5)	Echovirus 18	116 (4.2)
Echovirus 18	52 (2.5)	Echovirus 9	21 (5.7)	Parechovirus A3	15 (4.5)	Coxsackievirus B3	109 (4.0)
Echovirus 30	49 (2.4)	Coxsackievirus A9	19 (5.1)	Coxsackievirus A9	14 (4.2)	Echovirus 9	65 (2.4)
Parechovirus A3	43 (2.1)	Coxsackievirus B4	15 (4.1)	Coxsackievirus B2	10 (3.0)	Echovirus 11	64 (2.3)
Echovirus 9	41 (2.0)	Coxsackievirus B5	15 (4.1)	Echovirus 30	10 (3.0)	Parechovirus A3	62 (2.3)
Coxsackievirus B2	36 (1.8)	Echovirus 6	11 (3.0)	Coxsackievirus B1	9 (2.7)	Coxsackievirus B4	55 (2.0)
Coxsackievirus B5	32 (1.6)	Echovirus 25	10 (2.7)	Parechovirus A1	9 (2.7)	Coxsackievirus B5	53 (1.9)
Coxsackievirus A21	27 (1.3)	Coxsackievirus B3	9 (2.4)	Echovirus 11	8 (2.4)	Coxsackievirus B2	50 (1.8)
Enterovirus A71	23 (1.1)	Enterovirus D68	9 (2.4)	Coxsackievirus A10	7 (2.1)	Coxsackievirus A9	40 (1.5)
Coxsackievirus B4	22 (1.1)	Coxsackievirus A16	8 (2.2)	Coxsackievirus B5	6 (1.8)	Echovirus 6	40 (1.5)
Coxsackievirus A16	14 (0.7)	Coxsackievirus A5	6 (1.6)	Coxsackievirus A16	5 (1.5)	Echovirus 3	33 (1.2)
Echovirus 6	14 (0.7)	Coxsackievirus A10	5 (1.4)	Coxsackievirus A2	5 (1.5)	Coxsackievirus A16	27 (1.0)
—	—	Parechovirus A1*	5 (1.4)	—	—	Coxsackievirus A21*	27 (1.0)
**Total**	**1,984 (96.8)**	**Total**	**342 (92.4)**	**Total**	**308 (91.4)**	**Total**	**2,594 (94.1)**

* Additional types are shown where the least common type shown occurred with equal frequency.

EV-D68 was the most frequently reported type during 2014–2016, accounting for 1,542 (55.9%) reports for this period, including 1,395 (68.0%) in 2014, when a large nationwide outbreak of respiratory disease associated with EV-D68 occurred. In 2015, EV-D68 accounted for only nine (2.4%) reports that included virus type. EV-D68 again constituted a large percentage (40.9%) of reported types in 2016, but the 138 reports represented <10% of the EV-D68 reports in 2014. Overall, 1,351 (86.7%) EV-D68 detections were from respiratory specimens; 154 (9.9%) were from specimens whose source was unknown.

After EV-D68, the most frequently reported types during 2014–2016 were echovirus 30 (159; 13.1% of 1,216 reports of non–EV-D68 types), coxsackievirus (CV)-A6 (152; 12.5%), echovirus 18 (116; 9.5%), and CV-B3 (109; 9.0%). Among reports in which a type other than EV-D68 was detected (1,466), the most frequently reported specimen source was cerebrospinal fluid (493; 38.0% of 1,298 specimens with known source), followed by throat/nasopharyngeal swab (487; 37.5%).

Data reported to NREVSS were used to evaluate trends in the percentage of tests positive for EV over time. Among 62,210 specimens from which virus isolation was attempted in 47 laboratories, 0.6% (347) tested positive for untyped EV; among 70,915 specimens tested in 72 laboratories by reverse transcription–polymerase chain reaction, 5,555 (7.8%) tested positive. The percentage of specimens testing positive peaked in summer or early fall for all years (Figure 2). The decline in the percentage of positive results during July and August 2014 was associated with a substantial increase in the number of EV tests performed during the EV-D68 outbreak period.

**FIGURE 2:**
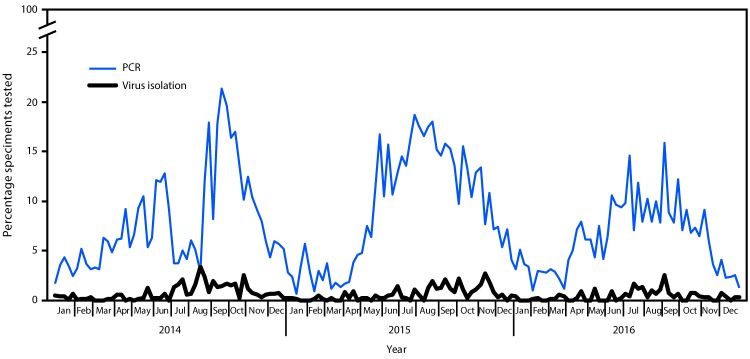
Percentage of specimens tested that were enterovirus-positive, by week and testing method used — National Respiratory and Enteric Virus Surveillance System, United States, 2014–2016 **Abbreviation:** PCR = polymerase chain reaction.

## Discussion

EV and PeV type surveillance in the United States was affected by the 2014 EV-D68 outbreak (*1*); this type accounted for 68% of identified types in 2014 and 56% of all reported types during 2014–2016. Increased vigilance and the need for rapid identification of new cases led to a large increase in diagnostic testing for EV and respiratory viruses among patients with respiratory illness during the late summer and autumn months of 2014. The number of reports with known type in 2014 was approximately three times higher than the 594 reports of EV and PeV in 2012, the year during the 2009–2013 period that witnessed the largest number of reports of typed EV and PeV (*2*,*3*).

The objectives of type-based EV and PeV surveillance in the United States are to 1) help public health practitioners determine long-term patterns of circulation for individual types, 2) interpret trends in picornavirus-associated illnesses by associating them with circulating types, 3) support recognition of disease outbreaks associated with circulating types, 4) guide the development of new diagnostic tests and therapies, and 5) monitor detections of poliovirus, which is nationally notifiable in the United States.

Reports to NESS continue to be affected by changes in diagnostic practices. For example, qualitative pan-EV molecular testing has largely replaced traditional cell culture virus isolation techniques in clinical settings because it produces results in a clinically relevant time frame and is more analytically sensitive (*4*). However, pan-EV molecular testing does not produce type-level results provided by viral culture, resulting in a lower frequency of reporting to NESS compared with prior decades (*4*). A CDC-developed real-time reverse transcription–polymerase chain reaction test for EV-D68 was widely adopted among public health laboratories in 2014. Qualitative pan-PeV testing is not as common as pan-EV testing in clinical laboratories in the United States, and PeV typing, for the most part, is limited to reference laboratories.

The findings in this report are subject to at least four limitations. First, NESS is a passive surveillance system that relies on voluntary reports from laboratories, and EV and PeV infections, except for polio, are not nationally notifiable in the United States. Second, to minimize the reporting burden for participating laboratories, patient-level clinical information is not routinely collected, so it is not possible to associate reported types with specific clinical manifestations or severity of illness. Third, typing tends to occur primarily during summer months, which might lead to underreporting of EV and PeV during other times of the year. Finally, although participating laboratories are encouraged to report monthly, reports are often delayed, making the timely compilation of data difficult.

Recent outbreaks, such as those of EV-D68–associated respiratory illness, CV-A6–associated severe hand, foot, and mouth disease, and a cluster of severe PeV-A3 infections among infants (*1*,*3*,*5*), highlight the continuing need for robust EV and PeV type surveillance. The associations between certain EV and PeV types and specific clinical manifestations have been well documented, but the epidemiology and associated clinical syndromes of many other EV and PeV types remain poorly characterized. Timely and robust type-based EV and PeV surveillance has the potential to inform disease prevention strategies by supporting the recognition of outbreaks and guiding the development of diagnostic tests and interventions. To do so would require improved maintenance and modernization of typing capacity within laboratories, timely and consistent reports from participating laboratories, and an increase in the number of reporting laboratories.

**Summary**

**What is already known about this topic?**

Enterovirus (EV) and parechovirus (PeV) infections can cause a variety of illnesses, ranging from asymptomatic infection to severe illness and death, and are divided into types.

**What is added by this report?**

During 2014–2016, reports of EV and PeV peaked in summer and early fall. Enterovirus D68 was the most frequently reported type (56%); echovirus 30, coxsackievirus A6, echovirus 18, and coxsackievirus B3 were also frequently reported.

**What are the implications for public health practice?**

Improved type-based surveillance can inform disease prevention strategies by supporting outbreak detection and guiding the development of new tests and interventions. Improving surveillance would require increasing the number and capacity of participating laboratories and timely reporting.
